# Supplementary material for: Calibrations of Low-Cost Air Pollution Monitoring Sensors for CO, NO2, O3, and SO2
Source: Sensors (Basel). 2021 Jan 2;21(1):256. doi: 10.3390/s21010256 (PMC7795951; doi:10.3390/s21010256)
Supplement: Supplementary file 1 [file sensors-21-00256-s001.pdf]

Supporting information

Calibrations of Low-Cost Air Pollution Monitoring Sensors for CO, NO_2, O_3, and SO_2

Pengfei Han [1,†], Han Mei [1,2,†], Di Liu [1,*], Ning Zeng [3], Xiao Tang [2], Yinghong Wang [2,*] and Yuepeng Pan [2]

[1] State Key Laboratory of Numerical Modeling for Atmospheric Sciences and Geophysical Fluid Dynamics, Institute of Atmospheric Physics, Chinese Academy of Sciences, Beijing 100029, China; pfhan@mail.iap.ac.cn (P.H.); meihan@mail.iap.ac.cn (H.M.)

[2] State Key Laboratory of Atmospheric Boundary Layer Physics and Atmospheric Chemistry, Institute of Atmospheric Physics, Chinese Academy of Sciences, Beijing 100029, China; tangxiao@mail.iap.ac.cn (X.T.); panyuepeng@mail.iap.ac.cn (Y.P.)

[3] Department of Atmospheric and Oceanic Science, and Earth System Science Interdisciplinary Center, University of Maryland, College Park, Maryland 20742, USA; zeng@umd.edu

* Correspondence: liudi@mail.iap.ac.cn (D.L.); wangyinghong@mail.iap.ac.cn (Y.W.)

† These authors contributed equally to this work and should be considered co-first authors.

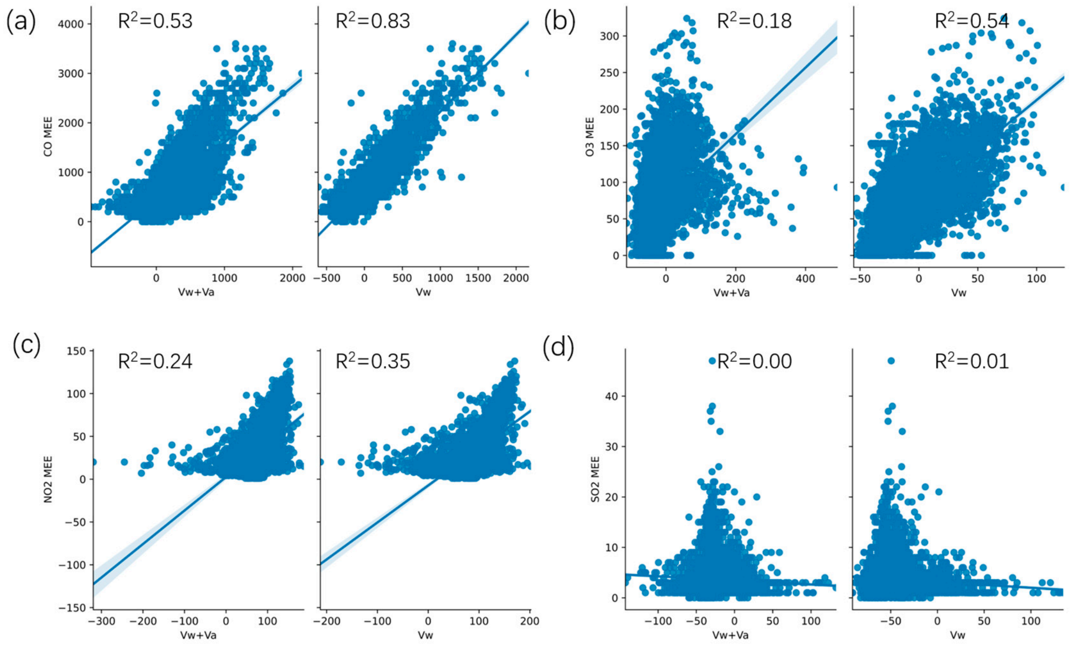

Figure S1. Scatter plot of sensor voltage signals (with Va and without Va) and MEE data. (**a**) CO, (**b**) O_3, (**c**) NO_2, (**d**) SO_2.

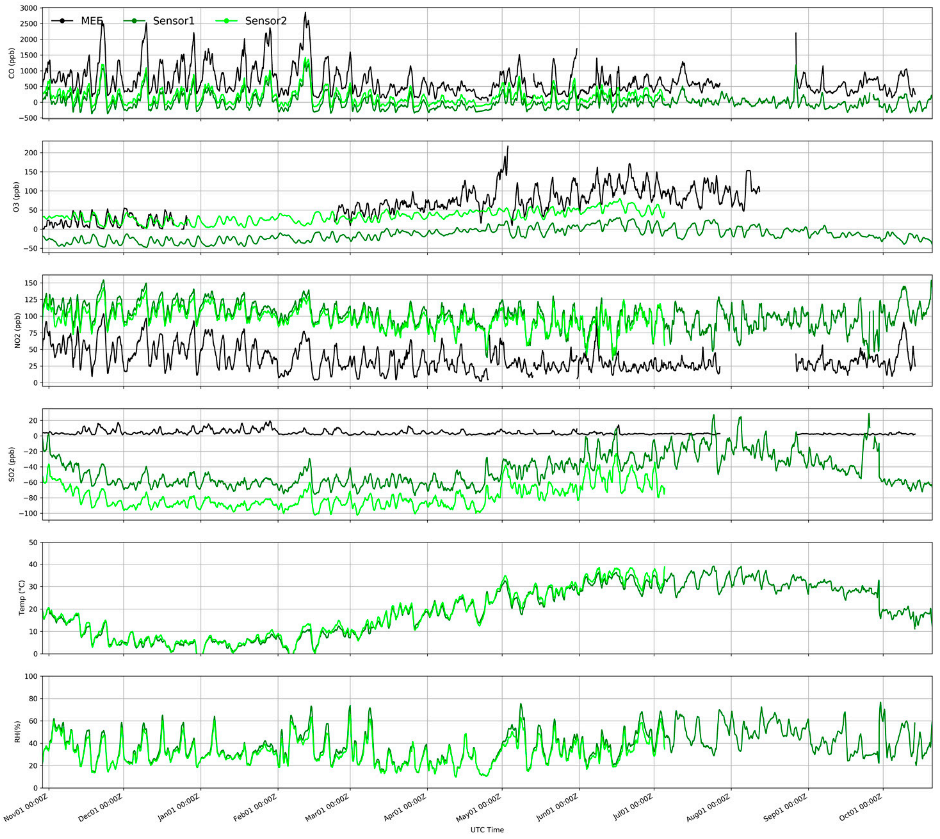

Figure S2. Comparison of (**a**) CO, (**b**) O_3, (**c**) NO_2, (**d**) SO_2 volume concentrations between the MEE Olympic Sports Center station (MEE) and the two uncalibrated sensor, (**e**) and (**f**) are temperature and relative humidity of the field where the sensor packages is located on 25 October 2019 to 10 October 2020, in Beijing based on a 24 h rolling average.

Table S1. Pairwise correlation coefficients(R^2) between sensor signals, environmental variables, and reference data.

Pollutant		MEE	Vw+Va	Vw	Va	Temp	RH
CO	MEE	1.00	**0.53**	**0.83**	0.05	0.06	0.21
	Vw+Va	0.53	1.00	0.68	0.12	0.06	0.23
	Vw	0.83	0.68	1.00	0.05	**0.02**	**0.26**
	Va	0.05	0.12	0.05	1.00	0.19	0.00
	Temp	0.06	0.06	0.02	0.19	1.00	0.00
	RH	0.21	0.23	0.26	0.00	0.00	1.00
O_3	MEE	1.00	**0.18**	**0.54**	0.08	0.40	0.01
	Vw+Va	0.18	1.00	0.71	0.00	0.25	0.25
	Vw	0.54	0.71	1.00	0.04	**0.51**	**0.19**
	Va	0.08	0.00	0.04	1.00	0.00	0.03
	Temp	0.40	0.25	0.51	0.00	1.00	0.00
	RH	0.01	0.25	0.19	0.03	0.00	1.00
NO_2	MEE	1.00	**0.24**	**0.35**	0.15	0.10	0.11
	Vw+Va	0.24	1.00	0.92	0.12	0.12	0.20
	Vw	0.35	0.92	1.00	0.25	**0.23**	**0.26**
	Va	0.15	0.12	0.25	1.00	0.06	0.24
	Temp	0.10	0.12	0.23	0.06	1.00	0.00

	RH	0.11	0.20	0.26	0.24	0.00	1.00
SO_2	MEE	1.00	**0.00**	**0.01**	0.00	0.02	0.01
	Vw+Va	0.00	1.00	0.35	0.07	0.26	0.05
	Vw	0.01	0.35	1.00	0.33	**0.49**	**0.02**
	Va	0.00	0.07	0.33	1.00	0.12	0.00
	Temp	0.02	0.26	0.49	0.12	1.00	0.00
	RH	0.01	0.05	0.02	0.00	0.00	1.00

Table S2. Performances of O_3 sensor with linear regressions (SLR, MLR).

linear regression calibration model	Train data		Test data	
	Before correction	After correction	Before correction	After correction
C_correction = a*C_raw+ b	R^2 = 0.54 RMSE = 85.36	R^2=0.54 RMSE = 36.08	R^2 = 0.59 RMSE = 84.44	R^2=0.59 RMSE = 34.21
C_correction =a* C_raw + b*Temp +c		R^2 = 0.56 RMSE = 35.17		R^2 = 0.64 RMSE = 31.97
C_correction = a*C_raw + b*RH + c		R^2=0.59 RMSE = 34.03		R^2=0.61 RMSE = 34.03
C_correction =a*C_raw+ b*Temp +c*RH + d		R^2=0.59 RMSE = 35.03		R^2=0.61 RMSE = 31.56

Table S3. Performances of NO_2 sensor with linear regressions (SLR, MLR).

linear regression calibration model	Train data		Test data	
	Before correction	After correction	Before correction	After correction
C_correction = a*C_raw+ b	R^2 = 0.35 RMSE = 71.50	R^2=0.35 RMSE = 20.91	R^2 = 0.44 RMSE = 71.56	R^2=0.44 RMSE = 20.35
C_correction =a* C_raw + b*Temp +c		R^2 = 0.35 RMSE = 20.89		R^2 = 0.44 RMSE = 20.37
C_correction = a*C_raw + b*RH + c		R^2=0.35 RMSE = 20.88		R^2=0.44 RMSE = 20.30
C_correction =a*C_raw+ b*Temp +c*RH + d		R^2=0.31 RMSE = 21.99		R^2=0.41 RMSE = 21.94

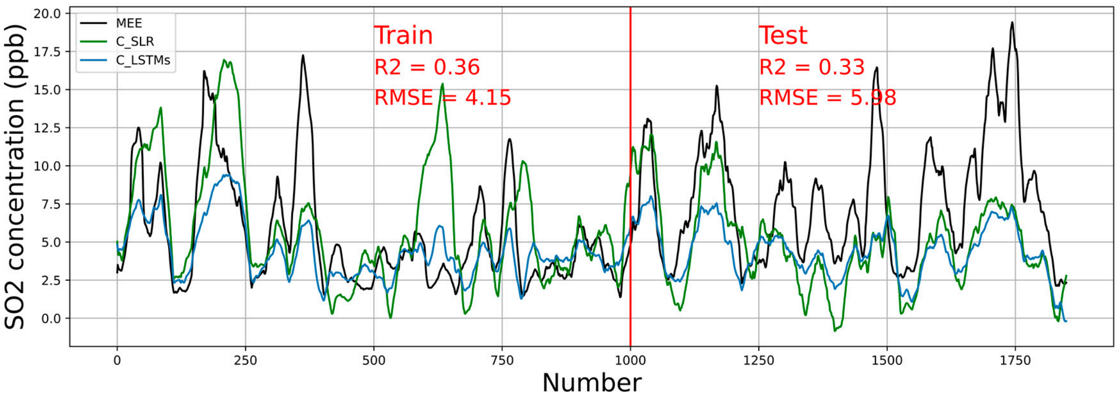

Figure S3. Time series in winter (December 2019–Feburary2020) of SO_2 sensor's SLR/LSTM calibrated data and MEE data based on a 24 h rolling average.

Table S4. Individual air quality index and corresponding pollutants volume concentration limit table in China.

Air quality level	CO (ppb)	O_3 (ppb)	NO_2 (ppb)	SO_2 (ppb)
Excellent	4366	82	53	57
Good	8732	102	106	191
light pollution	30563	153	372	248
moderate pollution	52395	204	638	305
heavy pollution	78592	408	1244	--

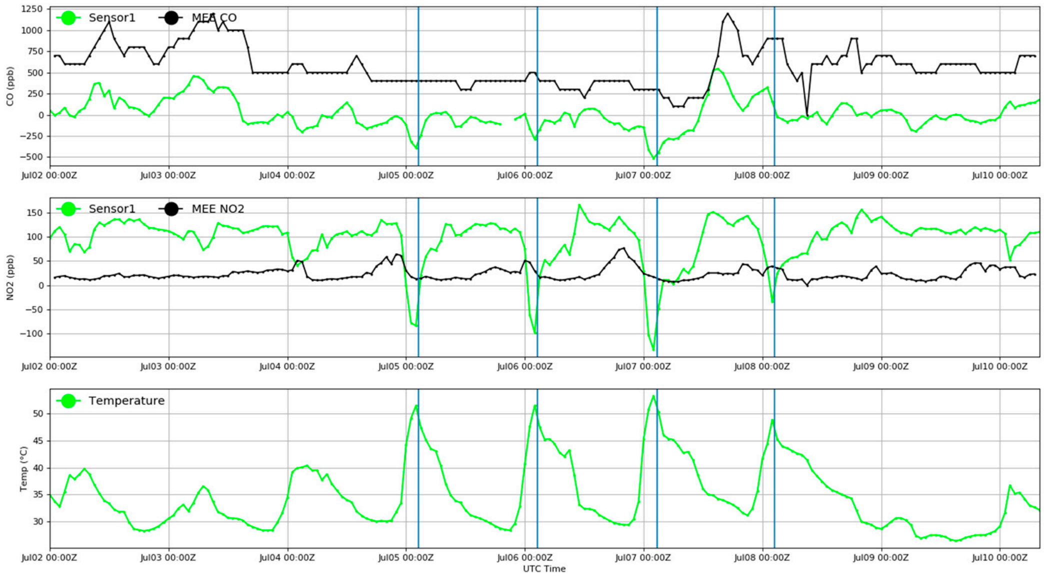

Figure S4. Time series of CO, NO_2 and temperature sensors and MEE during high temperature period in summer (2 July 2020–10 July 2020), the blue vertical line represents the time corresponding to the sensor temperature > 40 ℃.
